# New evidence for the effect of type 2 diabetes and glycemic traits on testosterone levels: a two-sample Mendelian randomization study

**DOI:** 10.3389/fendo.2023.1238090

**Published:** 2023-10-11

**Authors:** Chengyang Jiang, Yuwei Wang, Wenqiang Yang, Xinghai Yang

**Affiliations:** ^1^ Department of Pediatric Surgery, Maternal and Child Hospital of Hubei Province, Tongji Medical College, Huazhong University of Science and Technology, Wuhan, Hubei, China; ^2^ School of Medicine, Wuhan University of Science and Technology, Wuhan, Hubei, China

**Keywords:** Mendelian randomization, testosterone, diabetes, glycemic traits, sexual dysfunction

## Abstract

**Objective:**

Type 2 diabetes mellitus (T2DM) is an endocrine-related disease with an increasing incidence worldwide. Male sexual dysfunction is common in diabetic patients. Therefore, we designed a Mendelian randomization (MR) study to investigate the association of type 2 diabetes and 3 glycemic traits with testosterone levels.

**Methods:**

Uncorrelated single nucleotide polymorphisms (SNPs) associated with T2DM (N = 228), fasting insulin (N = 38), fasting glucose (N = 71), and HbA1c (N = 75) at the genome-wide significance were selected as instrument variables. Genetic associations with testosterone levels (total testosterone, TT, bioavailable testosterone, BT, and sex hormone-binding globulin, SHBG) were obtained from the UK Biobank studies and other large consortia. Two-sample MR analysis was used to minimize the bias caused by confounding factors and response causality. Multivariable MR analysis was performed using Body mass index (BMI), Triglycerides (TG), LDL cholesterol (LDL), and adiponectin to adjust for the effects of potential confounders.

**Results:**

Type 2 diabetes mellitus was associated with the decrease of total testosterone (β: -0.021,95%CI: -0.032, -0.010, p<0.001) and sex hormone binding globulin (β: -0.048,95%CI: -0.065, -0.031, p<0.001). In males, total testosterone (β: 0.058, 95% CI: 0.088, 0.028, p < 0.001) decreased. In females, it was associated with an increase in bioavailable testosterone (β: 0.077,95%CI: 0.058,0.096, p<0.001). Each unit (pmol/L) increase in fasting insulin was associated with 0.283nmol/L decrease in sex hormone-binding globulin (95%CI: -0.464, -0.102, p=0.002) and 0.260nmol/L increase in bioavailable testosterone (95%CI: -0.464, -0.102, p= 0.002). In males, sex hormone binding globulin decreased by 0.507nmol/L (95%CI: -0.960, -0.054, p= 0.028) and bioavailable testosterone increased by 0.216nmol/L (95%CI: 0.087,0.344, p= 0.001). In females, sex hormone binding globulin decreased by 0.714 nmol/L (95%CI: -1.093, -0.335, p<0.001) and bioavailable testosterone increased by 0.467nmol/L (95%CI: 0.286,0.648, p<0.001). Each unit (%) increase in HbA1c was associated with 0.060nmol/L decrease in sex hormone-binding globulin (95%CI: -0.113, -0.007, p= 0.026). In males, total testosterone decreased by 0.171nmol/L (95%CI: -0.288, -0.053, p=0.005) and sex hormone binding globulin decreased by 0.206nmol/L (95%CI: -0.340, -0.072, p=0.003). Total testosterone increased by 0.122nmol/L (95%CI: 0.012,0.233, p=0.029) and bioavailable testosterone increased by 0.163nmol/L (95%CI: 0.042,0.285, p=0.008) in females.

**Conclusions:**

Using MR Analysis, we found independent effects of type 2 diabetes, fasting insulin, and HbA1c on total testosterone and sex hormone-binding globulin after maximum exclusion of the effects of obesity, BMI, TG, LDL and Adiponectin.

## Introduction

With the improvement of nutritional status, type 2 diabetes mellitus has gradually become one of the major threats to human health (1). The number of adults with diabetes increased from 108 million to 422 million between 1980 and 2014 (2), with type 2 diabetes accounting for more than 90% of these cases (3). Diabetes has become one of the top three diseases in the world, and the global prevalence is increasing (4). Traditional cross-sectional studies suggest that gonadal function and testosterone levels are decreased in 33%-57% of patients with type 2 diabetes (5). At present, there are clinical studies on the intervention of blood glucose status in type 2 diabetic patients by exogenous testosterone supplementation (6). However, observational studies have limitations such as misclassification and reverse causality. At the same time, type 2 diabetes is a chronic metabolic disorder with a long course and early occult symptoms, so the causal relationship between type 2 diabetes and testosterone level is still unclear (7). There are gender differences in testosterone levels, and the effect of type 2 diabetes on testosterone levels in different genders is still unclear. The effects of various glycemic traits on testosterone levels are still controversial. The effect of different diabetes treatment regimens on altered testosterone levels is unknown. We aimed to investigate these issues by means of Mendelian randomization.

Mendelian randomization (MR) is an epidemiological method that enables causal inference by using single nucleotide polymorphisms (SNPs) as instrumental variables of exposure (8). Compared with observational studies, MR can reduce confounding bias because the genetic alleles are randomly aligned at conception and therefore not correlated with environmental and acquired factors. Because the genotype cannot be altered by disease, the MR design prevents reverse causality (9). Here, we performed a two-sample MR study to comprehensively examine the association of type 2 diabetes and glycemic traits (fasting insulin, fasting glucose, and HbA1c) with testosterone levels.

## Method

**Figure 1:**
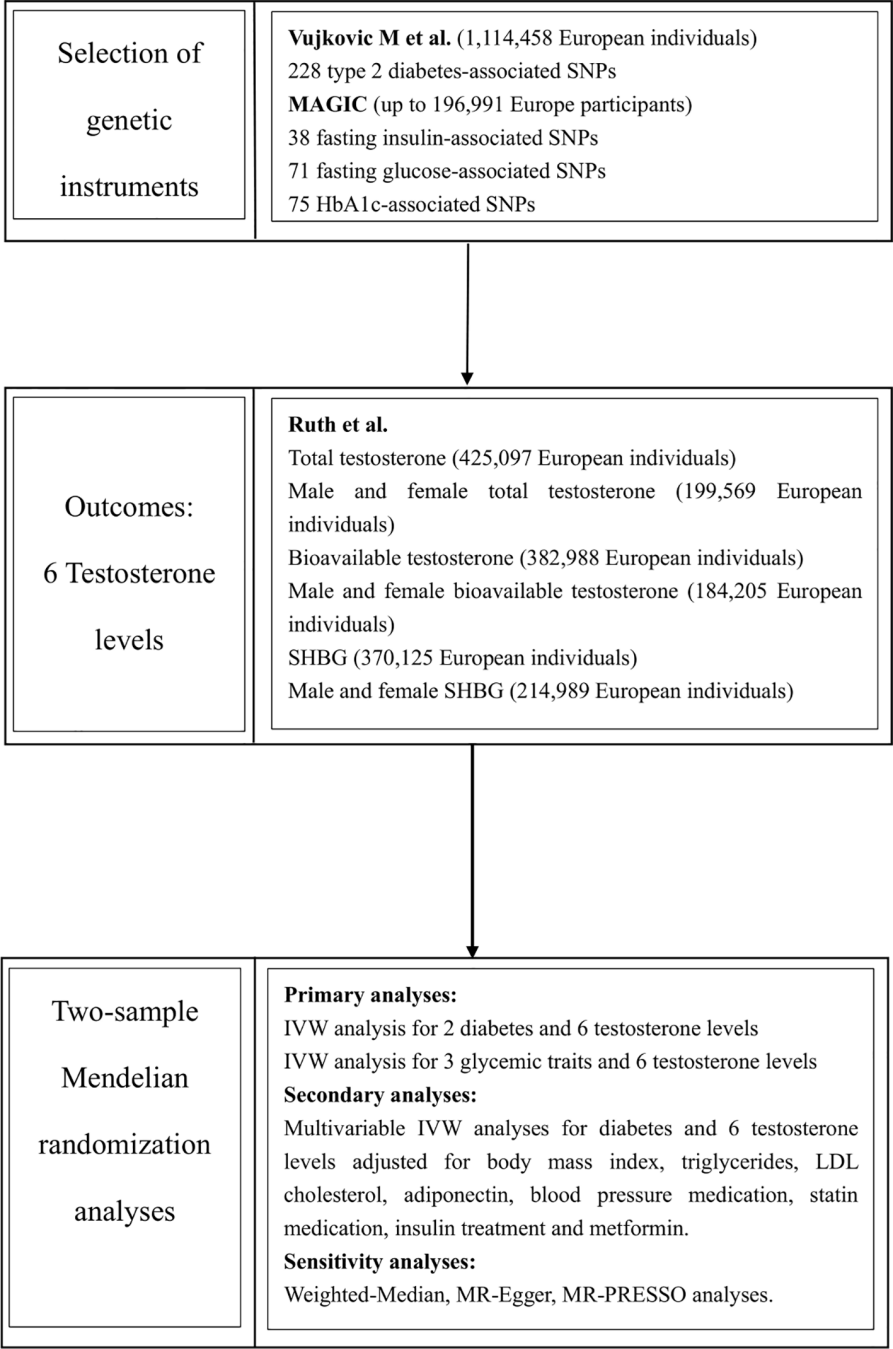
Study design. MAGIC, Meta-analyses of glucose and insulin-related traits consortium.

This two-sample MR analysis was designed to investigate type 2 diabetes and glycemic traits on testosterone levels, including total testosterone (TT), bioavailable testosterone (BT) and sex hormone-binding globulin (SHBG). Additional studies were conducted to investigate the effects of different diabetes treatment regimens on testosterone levels. This study was based on genome-wide association studies (GWAS) and publicly available data from the UK Biobank database and other large consortia, with no overlap between study populations. The included studies were approved by the relevant ethical review boards, and informed consent was obtained from participants (**Figure 1**).

### Selection of instrumental variables and data sources

Type 2 diabetes and glycemic traits (fasting insulin, fasting glucose, and HbA1c).

SNPs associated with type 2 diabetes and glycemic traits (fasting insulin, fasting glucose, and HbA1c) were selected from GWAS meta-analyses. These included 228,499 cases and 1,178,783 controls from the Million Soldier Retirement Program, DIAMANTE, Japan Bank of Biotechnology, and other studies (10). We selected SNPs with P<5*10^-8^ and calculated linkage disequilibrium between SNPs at each exposure by using the PLINK clustering method based on the 1000 Genomes European panel. SNPs with linkage disequilibrium (defined as R^2^ < 0.001 and clumping distance = 5000 kb) were excluded. Five SNPs that were also associated with obesity (11) and three SNPs associated with BMI were removed, leaving 228 SNPs to be used as instrumental variables for type 2 diabetes. 38 SNPs were used for fasting insulin, 71 SNPs for fasting glucose, and 75 SNPs for HbA1c.

### Body mass index

Pooled data on BMI were obtained from a meta-analysis (12) of 23 studies, which involves 32,161 individuals of European individuals whose populations did not overlap with the UK Biobank.

### Triglycerides and LDL cholesterol

Pooled data on TG and LDL were obtained from a meta-analysis (13) of 23 studies involving 94,595 individuals of European individuals, whose populations did not overlap with the UK Biobank.

### Adiponectin

The aggregate-level data on adiponectin were derived from a meta-analysis (14) of 16 studies involving 45,891 European individuals whose study populations did not overlap with the UK Biobank data.

### Medications for diabetes

A total of four different drugs were selected for analysis, namely insulin, metformin, blood pressure medication, and statin medication. Data on insulin and statin therapy were obtained from FinnGen, which included 218,792 European individuals (15, 16). Data on metformin and blood pressure medication were obtained from the UK Biobank, which included 462,933 and 249,710 European individuals, respectively (17, 18).

### Testosterone levels

SNPs for TT, BT, and SHBG levels were obtained from publicly available summary statistics (19). The database uses UK Biobank data, which includes phenotypic and biospecimens collected from approximately 500,000 individuals across the United Kingdom (20). Testosterone and SHBG levels (nmol/L) were measured in 230,454 and 189,473 participants, respectively, using a one-step competition assay and a two-step sandwich immunoassay. BT (nmol/L) was calculated from total testosterone and albumin, which was also measured by BCG analysis on a Beckman Coulter AU5800.

All data sources and instrumental variables are summarized in **Supplementary Tables 1**, **2**.

### Statistical analysis

Inverse-variance weighted (IVW) was used as the main MR method to estimate the causal relationship of type 2 diabetes and glycemic traits with testosterone levels. We used a random-effects model for all MR analyses because of the heterogeneity among the databases used. Simultaneously, sensitivity tests were also performed by weighted median, MR-Egger and MR-PRESSO to test the robustness of the results and verify horizontal pleiotropy. If more than 50% of the weights are derived from valid SNPs, the weighted median method can provide valid MR estimates. The embedded intercept of the MR-Egger regression analysis was used to detect horizontal pleiotropy and to provide correct estimates after correction for pleiotropy effects. Using MR-PRESSO to detect and correct for possible outliers, the MR-PRESSO global test can be used to estimate level pleiotropy caused by SNPs heterogeneity (21). Horizontal pleiotropy that could distort causal inferences was assessed by three sensitivity tests and the MR-Egger intercept test. The strength of IVs was assessed by calculating the F-statistic using the formula F= R^2^× (N −1 −K)/(1−R^2^) ×K, where R^2^ represents the proportion of variance in the exposure explained by the genetic variants, N represents sample size, and K represents the number of instruments (22). If the corresponding F-statistic was >10, it was considered that there was no significant weak instrumental bias (22). The power of the MR estimates was calculated using the online calculator tool (23) provided by Stephen Burgess (24).

Heterogeneity of VIs was calculated using Cochran’s Q statistic. At the same time, the potential sources of heterogeneity were identified by “leave-one-out” analysis for each SNPs.

Testosterone levels were defined as continuous variables, and β was used to assess the effect of type 2 diabetes and related glycemic measures on testosterone levels. All analyses were two-tailed. We used the R packages *TwoSampleMR* and *MRPRESSO* in R 4.1.3 for the analysis.

## Result

The study involved three different populations, i.e., patients with type 2 diabetes regardless of gender, male patients with diabetes and female patients with diabetes.

The relationship between the gene-predicted type 2 diabetes and testosterone level (nmol/L) was as follows (**Supplementary Table 3**).

Overall, type 2 diabetes was associated with decreased total testosterone (β: -0.021,95%CI: -0.032, -0.010, p<0.001) and sex hormone binding globulin (β: -0.048, 95%CI: -0.065, -0.031, p<0.001).

**Figure 2:**
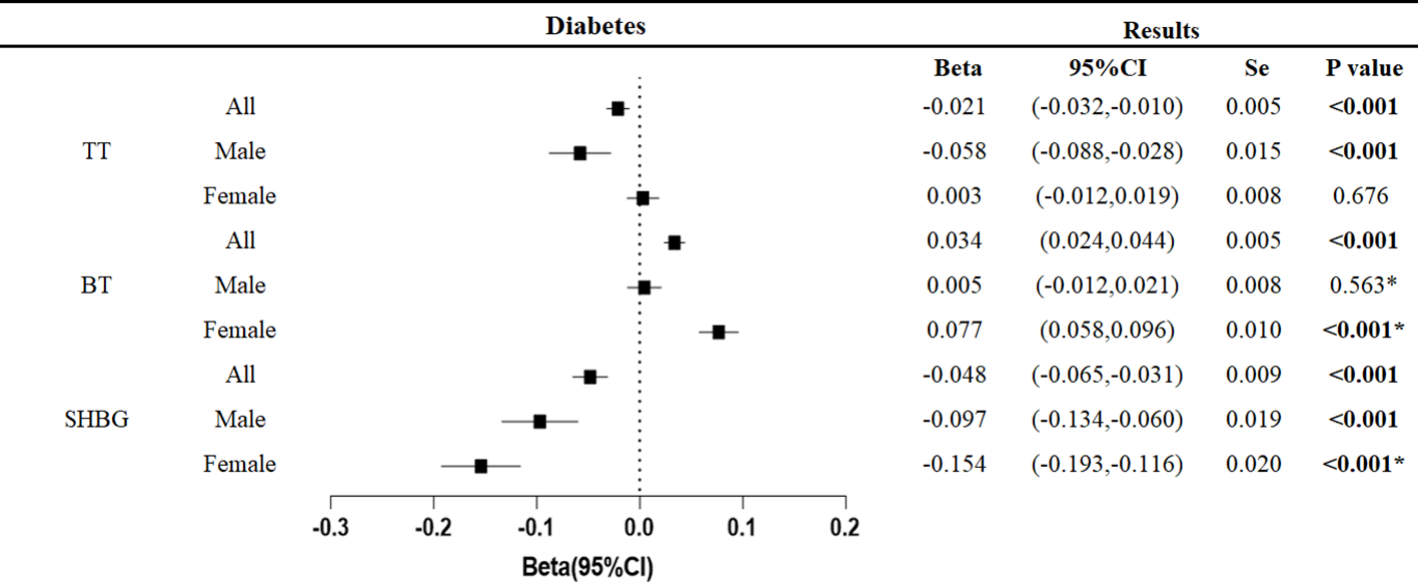
Associations of genetic liability to the type 2 diabetes mellitus with testosterone. P values are for ORs (95% CIs). Se is standard error. * The P value for pleiotropy is less than 0.05.

Similar trends were found in studies that differentiated between genders. In male patients with type 2 diabetes, total testosterone (β: -0.058, 95%CI: -0.088, -0.028, p<0.001) and sex hormone binding globulin (β: -0.097, 95%CI: -0.134, -0.060, p<0.001) decreased. In female patients with type 2 diabetes, sex hormone binding globulin (β: -0.154, 95%CI: -0.193, -0.116, p<0.001) decreased, but bioavailable testosterone (β: 0.077, 95%CI: 0.058, 0.096, p<0.001) showed the opposite trend (**Figure 2**).

Pleiotropy analysis showed pleiotropic effects in male bioavailable testosterone, female bioavailable testosterone, and female sex hormone-binding globulin. This persisted even after exclusion of outliers by MR-PRESSO test and hence making the results of these three studies unreliable. We excluded these indicators in the analyses below. All but the three studies did not detect pleiotropy, and the results were robust with narrowed confidence intervals after exclusion of outliers by MR-PRESSO testing.

The results were robust after adjustment for BMI, TG, LDL, and adiponectin, with no significant change in the overall trend. The results are summarized in the **Supplementary Table 4**.

### Glycemic traits and testosterone levels

**Figure 3:**
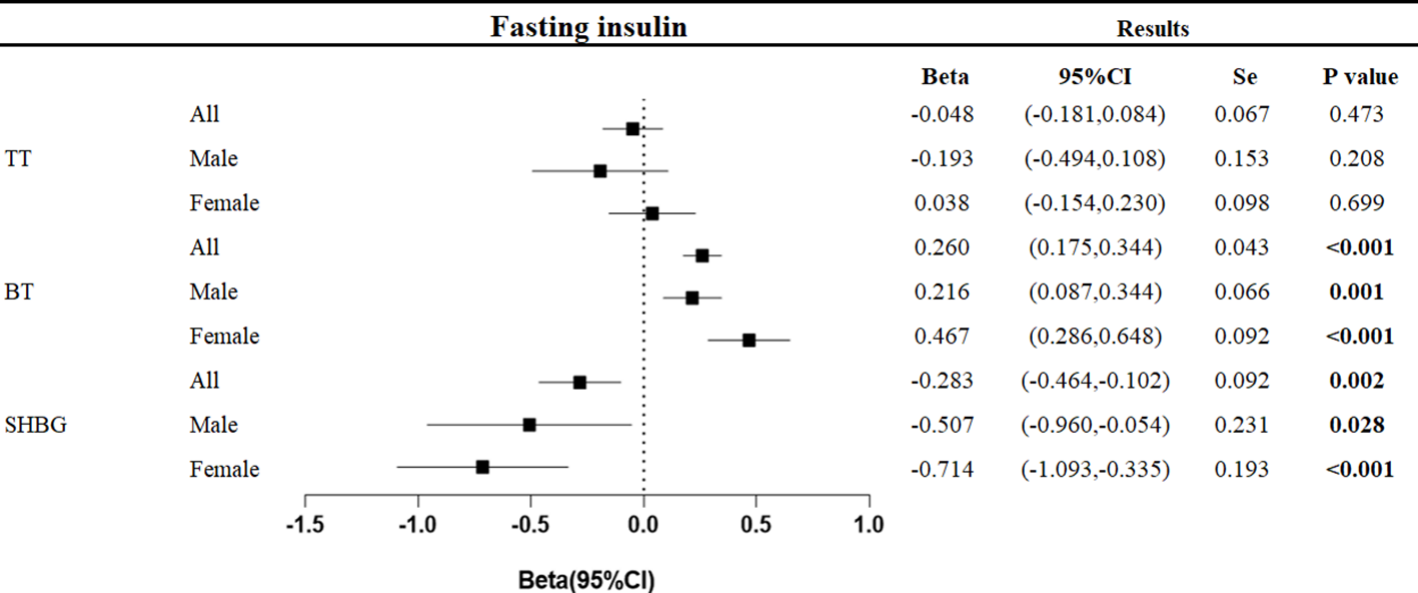
Associations of genetic liability to fasting insulin with testosterone. Beta is the relationship between per unit increase in fasting insulin (pmol/L) and testosterone level (nmol/L). Se is standard error. P values are for ORs (95% CIs). * The P value for pleiotropy is less than 0.05.

The relationship between the gene-predicted increase in fasting insulin level per unit (pmol/L) and testosterone level (nmol/L) was as follows (**Figure 3**).

At the overall level, each unit increase in fasting insulin was associated with a decrease in sex hormone-binding globulin of 0.283 nmol/L (95%CI: -0.464, -0.102, p=0.002) and an increase in bioavailable testosterone of 0.260 nmol/L (95%CI: 0.175,0.344, p<0.001).

In men, each unit increase in fasting insulin was associated with a decrease in sex hormone-binding globulin of 0.507 nmol/L (95%CI: -0.960, -0.054, p=0.028) and an increase in bioavailable testosterone of 0.216 nmol/L (95%CI: 0.087, 0.344, p<0.001). In women, each unit increase in fasting insulin level was associated with a 0.714 nmol/L decrease in sex hormone-binding globulin (95%CI: -1.093, -0.335, p<0.001) and a 0.467 nmol/L increase in bioavailable testosterone (95%CI: 0.286, 0.648, p<0.001).

**Figure 4:**
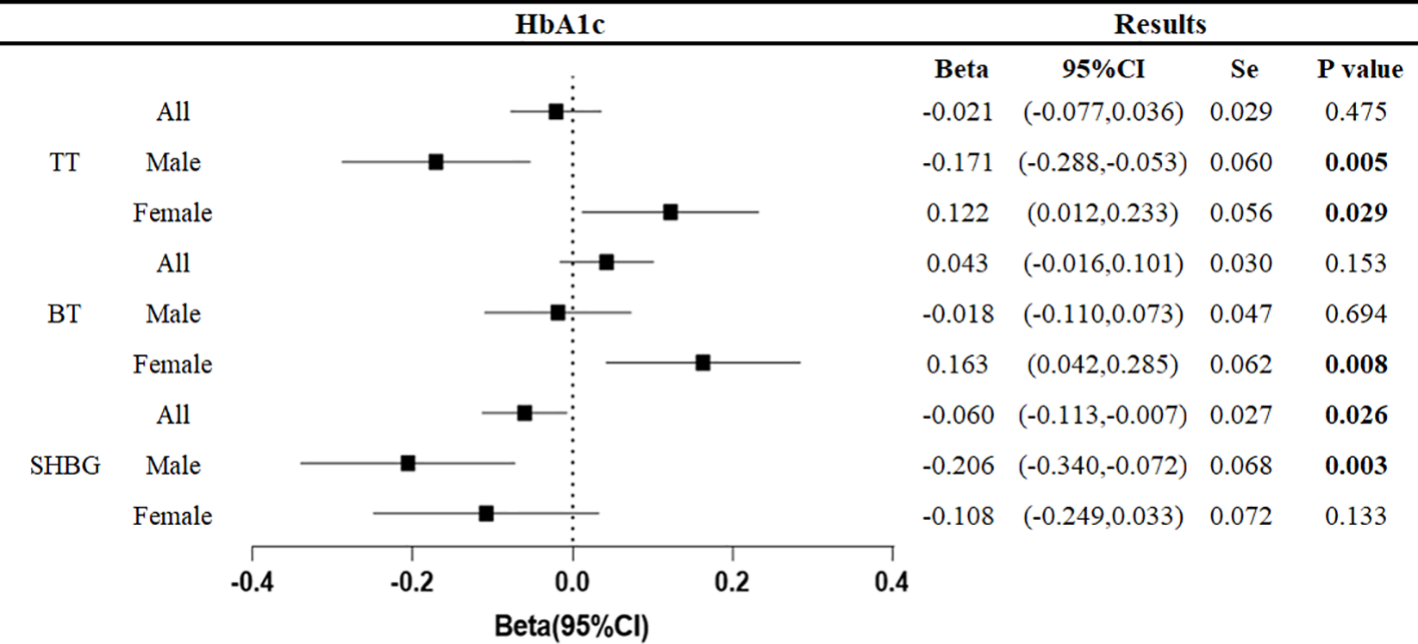
Associations of genetic liability to HbA1c with testosterone. Beta is the relationship between per unit increase in HbA1c (%) and testosterone level (nmol/L). Se is standard error. P values are for ORs (95% CIs). * The P value for pleiotropy is less than 0.05.

The relationship between the genetically predicted increase in HbA1c per unit (%) and testosterone level (nmol/L) was as follows (**Figure 4**).

At the overall level, each unit increase in HbA1c was associated with a 0.060 nmol/L decrease in sex hormone binding globulin (95%CI: -0.113, -0.007, p=0.026).

In men, each unit increase in HbA1c was associated with a decrease in total testosterone of 0.171 nmol/L (95%CI: -0.055, -0.053, p=0.005) and sex hormone-binding globulin of 0.206 nmol/L (95%CI: -0.340, -0.072, p=0.003). In women, each unit increase in HbA1c was associated with 0.122 nmol/L (95%CI: 0.012,0.233, p=0.029) increase in total testosterone and 0.163 nmol/L (95%CI: 0.042,0.285, p=0.008) increase in bioavailable testosterone.

**Figure 5:**
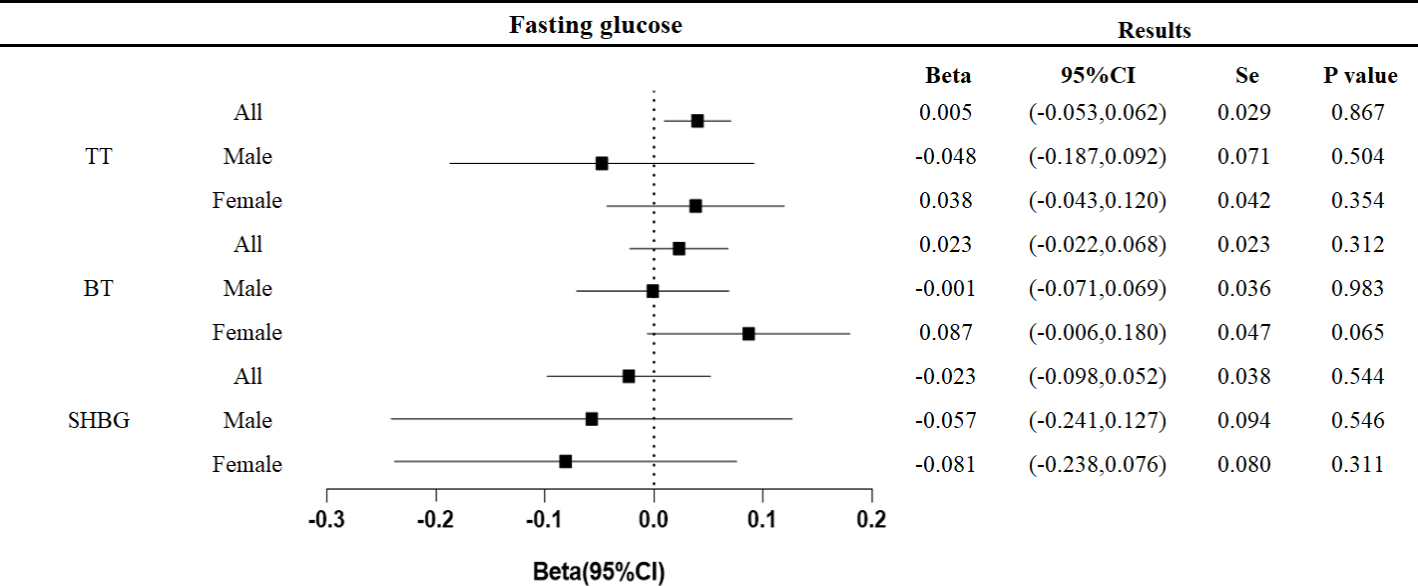
Associations of genetic liability to fasting glucose with testosterone. Beta is the relationship between per unit increase in fasting glucose (nmol/L) and testosterone level (nmol/L). Se is standard error. P values are for ORs (95% CIs). * The P value for pleiotropy is less than 0.05.

No effect of fasting glucose on testosterone levels was found in this study (**Figure 5**).

No pleiotropic effects were found in any of the above studies, and the confidence intervals of the results were narrowed after exclusion of outliers by the MR-PRESSO test. The results are summarized in the **Supplementary Tables 5**–**7**.

### Medications for diabetes

Multivariate Mendelian randomization analysis was performed for the four drugs (insulin, metformin, blood pressure medication, and statin medication). After adjustment for insulin therapy, the difference in testosterone levels (including total testosterone, bioavailable testosterone, and sex hormone-binding globulin) disappeared in all patients with diabetes, regardless of gender. At the same time, the downward trend of testosterone level was curbed, but showed an upward trend. Although this difference was not statistically significant, we can at least assume that the hypo-testosterone state was improved with insulin treatment. After adjusting for metformin treatment, the overall difference in testosterone levels in diabetic patients disappeared, except for bioavailable testosterone and sex hormone binding globulin levels in female patients. However, the results were not significantly altered after adjustment for statin therapy and antihypertensive medication alone. We summarized the relevant results in the **Table 8**.

## Discussion

Testosterone, an endogenous hormone, is the main male hormone produced by the testis and the precursor of estrogen synthesized by the female ovary (25). In males, it is mainly responsible for promoting the development and maintenance of male sexual characteristics (26) and plays a significant role in the development and maintenance of the male skeletal system (27). It is responsible for the maintenance of sexual desire in females (28). In general, low testosterone is thought to be closely related to sexual dysfunction (29). Testosterone not only has a major effect on sexual function, but also has effects on metabolism, mood, and cognition (30, 31).

Many observational studies have shown that testosterone levels in type 2 diabetic patients are lower than those in normal subjects (32). Due to the limitations of observational studies, causality cannot be fully determined, and confounding factors cannot be well excluded from studies. The study is the first to examine this issue using genetic tools. We investigated this issue using a large sample of diabetes GWAS data and three glycemic traits as exposure factors.

Fasting glucose represents short-term blood glucose, and HbA1c represents blood glucose up to three months (33). We also used the fasting insulin level as an exposure factor, which provides a proxy for the degree of insulin resistance (34). Testosterone levels vary greatly between men and women (35), so we also conducted sex-specific studies. In addition, we also adjusted for common diabetes drugs and obtained the improvement of testosterone decline with various treatment regimens.

Testosterone levels represent the sum of unbound as well as bound testosterone in the circulation. Most circulating testosterone is bound to SHBG and albumin, and only 2.0% to 4.0% of circulating testosterone is unbound or free (36). Bioavailable testosterone was defined as the sum of testosterone bound to albumin and free testosterone. Total testosterone was numerically equivalent to the sum of bioavailable testosterone and SHBG-bound testosterone. At the same time, the free hormone hypothesis states that the intracellular concentration and biological activity of hormones depend on the concentration of free hormones and not on the hormones in plasma bound to proteins (37).

The association between type 2 diabetes and testosterone levels has previously been questioned for two reasons. First, cross-sectional studies suggest a strong inverse association between testosterone and obesity (38), which is often accompanied by abnormal lipid metabolism in patients with diabetes. Therefore, it is not clear whether the low testosterone status in diabetic patients is affected by abnormal lipid metabolism. Second, patients with type 2 diabetes always have lower SHBG levels (39). Testosterone levels are largely affected by SHBG, especially as some studies have found no significant difference in bioavailable testosterone levels in patients with diabetes (40), which contrasts with the prevalence of sexual dysfunction in patients with diabetes. These problems are due to the nature of observational studies, which cannot well rule out confounding.

To address the first question, we found that the effect of diabetes on testosterone was still significant after excluding obesity-related genes and adjusting for BMI, lipid parameters (TG and LDL) and serum adiponectin. Especially for male patients, it can be determined that their total testosterone and sex hormone binding globulin showed a statistically significant decrease. Clear results were obtained after minimizing the effect of lipids and obesity. This effect is probably due to hyperglycemia and insulin resistance (41) due to diabetes itself and is independent of diabetic dyslipidemia. The analysis of three glucose traits also provided better support for this conclusion.

To address the second issue, in this study, three different indices of testosterone level were analyzed separately. Although a statistically significant increase in bioavailable testosterone was found in total and in women, this conclusion was not robust due to pleiotropy. Therefore, our study cannot support an increase in bioavailable testosterone levels in patients with diabetes, especially in studies that distinguish between sex. We suspect that the combination of hyperglycemia and insulin resistance is responsible for complex changes in bioavailable testosterone levels that require further elucidation. But in any case, there is no doubt that total testosterone and SHBG levels are significantly decreased in diabetic patients.

It is important that we obtain more valuable results after adjustment for conventional medications in the treatment of diabetes. Blood pressure medication and statin medication did not affect testosterone levels. However, our results were significantly changed after adjustment for exogenous insulin injection and oral metformin treatment. Especially after insulin injection treatment, the three indicators of testosterone level showed an opposite upward trend. In this study, we found that an increase in fasting insulin measures was associated with a decrease in testosterone levels, particularly sex hormone-binding globulin. Therefore, it can be concluded that injection of exogenous insulin after the appearance of insulin resistance can still have a positive effect on the low testosterone status caused by diabetes. This is also consistent with the clinical observation (42).

For the treatment of diabetes, the current guidelines of most countries do not recommend direct injection of exogenous insulin. Our results suggest that oral administration of metformin and injection of exogenous insulin may be a good way to improve low testosterone status in diabetic patients.

Previously, animal models of diabetes and metabolic syndrome have shown that hyperglycemia and insulin resistance play an important role in causing hypogonadism (43, 44). Animal studies confirm that mice with insulin receptor knockout exhibit hypogonadotropic hypogonadism (45, 46). Cellular studies in rat hypothalamic neurons have found that insulin plays a key role in promoting GnRH secretion (47). In addition to its action on the hypothalamus, insulin also acts directly on the liver to stimulate SHBG production (48, 49).

It has also been noted that lifestyle or pharmacologic management that improves insulin resistance increases testosterone levels (5) It has also been suggested that testosterone deficiency is associated with impaired gonadotropin response (50). It has been found that long-term hyperglycemia may lead to metabolic imbalance, inflammation, and oxidative stress, which may lead to a decrease in testosterone level (51).

Our analysis was performed according to gender in our study, and the changes in indicators of female patients often showed opposite trends compared with those of male patients, especially in total testosterone and bioavailable testosterone. This is consistent with what we have observed clinically (52). We speculate that it may be related to the fact that physiological amounts of estrogen in women reduce insulin resistance (53).

In conclusion, we used Mendelian randomization to determine the effects of T2DM, fasting insulin, and HbA1c on the three testosterone levels after controlling for SNPs associated with obesity and BMI and adjusting for confounding factors such as BMI, TG, LDL, and serum adiponectin. Compared with fasting plasma glucose, fasting insulin and Hb1Ac were more powerful predictors of SHBG and total testosterone. Previously, the causal relationship between the changes of sex hormones in diabetic patients has been controversial. Combined with the characteristics of Mendelian randomization studies, we believe that our study can solve this problem to a certain extent. We also found that oral metformin and injection of exogenous insulin improved the low testosterone status in diabetic patients after adjusting the common treatment methods.

## Limitations of our study

First, although we excluded the currently known FTO gene and performed reanalysis with adjustment for BMI, TC, LDL, and serum adiponectin, we could not completely rule out the effect of obesity on testosterone levels. Second, limited by GWAS, we decided to use fasting insulin levels as a proxy for insulin resistance. Although this indicator can represent insulin resistance to a certain extent, it is not the gold standard of insulin resistance (54), and it will also introduce some bias. Third, we used SNPS pooled from the meta-analysis, which makes it difficult to calculate f-values, so we cannot completely rule out the effect of weak instrumental variables. Fourth, different measures of testosterone levels can yield different results (55). Fifth, the pleiotropic effect found in the analysis of biologically available testosterone levels is fatal to the reliability of the results of the Mendelian randomization analysis. We subsequently added analyses of different glycemic measures, but more reliable methods are needed.

## Data availability statement

The original contributions presented in the study are included in the article/**Supplementary Material**. Further inquiries can be directed to the corresponding author.

## Author contributions

CJ: Conceptualization, Methodology, Software, Investigation, Formal Analysis, Writing - original draft. YW: Data Curation, Writing - original draft. WY: Visualization, Investigation. XY: Conceptualization, Funding acquisition, Resources, Supervision, Writing - review & editing. All authors contributed to the article and approved the submitted version.
